# *Candida albicans* Mrv8, is involved in epithelial damage and biofilm formation

**DOI:** 10.1093/femsyr/foaa033

**Published:** 2020-06-25

**Authors:** Anna Carolina Borges Pereira Costa, Graziella Nuernberg Back-Brito, François L Mayer, Bernhard Hube, Duncan Wilson

**Affiliations:** Department of Biosciences and Oral Diagnosis, São Paulo State University (Unesp), Institute of Science and Technology (ICT); São José dos Campos, Brazil; Department of Microbial Pathogenicity Mechanisms, Hans-Knoell-Institute, Jena, Germany; Aberdeen Fungal Group, School of Medicine, Medical Sciences and Nutrition, University of Aberdeen, Institute of Medical Sciences, Aberdeen, United Kingdom; Department of Biosciences and Oral Diagnosis, São Paulo State University (Unesp), Institute of Science and Technology (ICT); São José dos Campos, Brazil; Department of Microbial Pathogenicity Mechanisms, Hans-Knoell-Institute, Jena, Germany; Department of Microbial Pathogenicity Mechanisms, Hans-Knoell-Institute, Jena, Germany; Friedrich Schiller University, Jena, Germany; Department of Microbial Pathogenicity Mechanisms, Hans-Knoell-Institute, Jena, Germany; Aberdeen Fungal Group, School of Medicine, Medical Sciences and Nutrition, University of Aberdeen, Institute of Medical Sciences, Aberdeen, United Kingdom; Medical Research Council Centre for Medical Mycology, School of Biosciences, University of Exeter, Stocker Rd, Exeter EX4 4QD, Exeter, United Kingdom

**Keywords:** *Candida albicans*, biofilm, Marvel family, caspofungin, host-pathogen interaction, calcium homeostasis

## Abstract

*Candida albicans* is the most common human fungal pathogen that can cause superficial and deep-seated infections in susceptible individuals. Despite its medical importance, the vast majority of *C. albicans* genes remain of unknown function. Here, we report a role for the lineage-specific gene, *MRV8*, in host pathogen interactions, mycelial microcolony maturation and biofilm formation. *In silico* analysis indicated that *MRV8* encodes a four-pass transmembrane protein unique to the closely related pathogens *C. albicans* and *Candida dubliniensis*. Deletion of *MRV8* did not affect *C. albicans* adherence to, or initial invasion into human oral epithelia, but inhibited mycelial development and strongly reduced epithelial damage. *mrv8*Δ/Δ cells exhibited a media-dependent defect in biofilm formation and mutant biofilm metabolic activity was enhanced by cyclosporin A. *mrv8*Δ/Δ biofilms were more tolerant to treatment with caspofungin, but not to fluconazole or amphotericin B. Co-stimulation with calcium chloride and calcofluor white rescued biofilm growth in the presence of caspofungin, and this rescue-effect was Mrv8-dependent. Together, our data demonstrate an important role for a lineage-specific gene (*MRV8*) in *C. albicans* biofilm formation, drug tolerance and host–pathogen interactions.

## Teaser

Mrv8 is involved in mycelial maturation, epithelial damage and biofilm-associated caspofungin tolerance.

## INTRODUCTION

*Candida albicans* is normally a commensal member of the human microbiota (mycobiota) and can cause disease when an imbalance in host immunity or a disturbance of the normal microbiota composition allows the transition to a pathogenic lifestyle (Gow and Hube 2012). The pathogenicity of this fungus is mediated by a number of factors including adhesion to host cells, invasion and damage, which is predominantly caused by secretion of the cytolytic toxin, candidalysin (Moyes *et al*. 2016). Biofilms also play an important role in pathogenicity as they can serve as reservoirs, driving recurrent candidaemia.

Biofilms are surface-associated communities of cells encased within an extracellular matrix. Biofilm development emerges in several phases and can be divided into distinct stages. In early stages, yeast cells adhere to the substratum forming the biofilm basal layer, followed by co-aggregation of cells and colonization. The intermediate stage is characterized by the formation of hyphae and pseudohyphae and these filamentous cells continue to develop to the mature stage when the biofilm exhibits a three-dimensional structure composed of all three morphological cell types embedded in extracellular matrix material. Finally, disperser cells can be released from the biofilm to reinitiate the process, or serve as an infection reservoir (For reviews, see: Mathe and Van Dijck 2013; Desai, Mitchell and Andes 2014; Ramage, Robertson and Williams 2014; Nobile and Johnson 2015; Gulati and Nobile 2016; Soll and Daniels 2016).

*Candida albicans* biofilms can grow on host tissues, such as epithelia and on abiotic medical and dental devices. The organization of the biofilm structure protects its inhabitants from attack by host immunity and biofilms can exhibit extremely high resistance to antifungal drugs. Indeed, *C. albicans* biofilms can exhibit 30 to 200 times higher antifungal drug resistance than planktonic cells (Hawser and Douglas 1995). Biofilm cells are intrinsically resistant to fluconazole and sensitive only to high concentrations of amphotericin B. On the other hand, echinocandins, which inhibit cell wall β-glucan synthesis, and amphotericin B lipid complexes are more effective at lower concentrations (Chandra *et al*. 2001; Kuhn *et al*. 2002; Lazzell *et al*. 2009; Uppuluri *et al*. 2011). The mechanisms of biofilm antifungal resistance are not yet fully understood, but the resistant phenotype has been linked to overexpression of drug targets, higher cell density, heterogeneous populations of cells with different growth rates, expression of efflux pumps (CDR1–2 and MDR1), extracellular matrix (especially β-glucan), which sequesters antifungal drugs, subpopulations of persister cells, and tolerance mediated by the molecular chaperone Hsp90 through regulation of the calcineurin pathway and its downstream transcription factor, Crz1 (Chandra *et al*. 2001; Nett *et al*. 2007; Singh *et al*. 2009; Nett *et al*. 2011; Robbins *et al*. 2011; Ramage *et al*. 2012; Taff *et al*. 2012; Tobudic *et al*. 2012; Mathe and Van Dijck 2013).

Mrv8 (orf19.3908, C5_04250W) is a member of the MARVEL (*MA*L and related proteins for vesicles trafficking and membrane *l*ink) domain protein family. This family consists of eight members and orthologues also exist in *C. dubliniensis*. Douglas, Wang and Konopka (2013) reported that another family member, Nce102, was involved in invasive growth, but did not find altered phenotypes for the other family members. Our own group reported that *mrv8*Δ/Δ was defective for epithelial damage (Wilson *et al*. 2014).

Here, we report a role for *C. albicans* Mrv8 in epithelial damage, mycelial development, biofilm formation and caspofungin (CSF) tolerance.

## MATERIALS AND METHODS

### Strain construction

For generation of a *mrv8Δ/Δ+MRV8* complemented strain, a sequence containing the open reading frame and 376 base pairs upstream and 282 base pairs downstream sequences were amplified from SC5314 *C. albicans* genomic DNA using the Phusion High Fidelity DNA Polymerase Kit (Finnzymes). The PCR product was purified with the QIAquick PCR Purification kit (Qiagen) and then cloned into the Pcr2.TOPO vector (Invitrogen). The plasmid was digested with *Sall* and *MluI*, following gel extraction with the QIAquick Gel Extraction kit (Qiagen). The plasmid CIp10 was also digested with *SalI* and *MluI* and the restriction enzymes were heat inactivated by incubation at 65°C/20 min. Next, the linearized plasmid was dephosphorylated with calf intestinal alkaline phosphatase (New England BioLabs) and gel extracted using the QIAquick Gel Extraction kit (Qiagen). The *MRV8* insert and CIp10 plasmid were ligated at 25°C/30 min using the Quick Ligation Kit (New England BioLabs). Five µl of the ligation production was used to transform *Escherichia coli* DH5α and the positive clones were selected on LB agar [1% bacto-tryptone, 0.5% yeast extract, 1% NaCl, 2% agar] plates supplemented with 50 µg ml^−1^ ampicillin. The plasmid carrying the insert was re-isolated using the miniprep (Peqlab) and midiprep (Qiagen) kit and confirmed by control digestions with *StuI*, *NcoI*, and *MfeI*. The plasmid was digested with *StuI* prior the transformation into the uridine auxotrophic *C. albicans* strain *mrv8Δ/Δura^−^*. Positive clones were selected on SD agar [2% glucose, 0.17% yeast nitrogen base, 0.5% ammonium sulfate, 2% agar] plates without amino acids and correct integration was verified by PCR using primers RPF-F1/URA3-F2 and MRV8-INT-F/RPF1-R1. The strains and primers used in this study are listed in Table 1 and Table 2, respectively.

**Table 1.** *C. albicans* strains used in this study.

Strain	Genotype	Reference
BWP17 + CIp30	*ura3*::Δ/Δ*imm434/ura3*::Δ/Δ*imm434 arg4*::*hisG/arg4*::*hisG his1*::*hisG/his1*::*hisG + *CIp30	Mayer *et al*. (2012)
*mrv8Δ/Δ*	*orf19.3908∆::ARG4/orf19.3908∆::HIS1 + CIp10 (URA3)*	Wilson *et al*. (2014)
*mrv8Δ/Δ::MRV8*	*orf19.3908∆::ARG4/orf19.3908∆::HIS1 + CIp10 (ORF19.3908, URA3)*	This study

**Table 2.** Primers used in this study.

*Primer*	Sequence	Reference
MRV8-Rec-F	GTCGACAGTAATCTATACATTACACAACC	This study
MRV8-Rec-R	ACGCGTATACCATCAGGGATGTTGC	This study
MRV8-Int-F1	GTTGGGCTATTGGCGTTATTTGTG	This study
RPF-F1	GAGCAGTGTACACACACACATCTTG	Wilson *et al*. (2014)
RPF1-R1	CGCCAAAGAGTTTCCCCTATTATC	This study
URA3-F2	GGAGTTGGATTAGATGATAAAGGTGATGG	Gola *et al*. (2003)
HIS-F2	GGACGAATTGAAGAAAGCTGGTGCAACCG	Gola *et al*. (2003)
HIS-R2	CAACGAAATGGCCTCCCCTACCACAG	Gola *et al*. (2003)
ARG4-F2	GGATATGTTGGCTACTGATTTAGC	Martin *et al*. (2011)
ARG4-R2	AATGGATCAGTGGCACCGGTG	Gola *et al*. (2003)

### Biofilm development

Biofilms were grown as described previously (Nobile and Mitchell 2005; Junqueira *et al*. 2011). The strains were cultivated in YPD [1% yeast extract, 2% bacto-peptone, 2% D-glucose] at 30°C/24 h. The cultures were adjusted to OD_600_ at 0.5 in Spider medium [1% nutrient broth, 1% mannitol, 0.4% K_2_HPO_4_, pH 7.2] and 250 µl was added to the wells of a 96-well flat-bottom microtiter plate (TPP) followed by incubation at 37°C for 1.5 h with shaking at 75 rpm (Quimis). After washing the wells, fresh medium was added and the plate was incubated at 37°C/48 h at 75 rpm. The medium was changed every 24 h.

The protocol for XTT colorimetric assay was described by Jin *et al*. (2003). Briefly, the biofilms were washed twice and treated with 197.5 µl of PBS, 50 µl of XTT (1 mg mL^−1^) (Sigma-Aldrich) and 2.5 µl of menadione (0.4 mM) (Sigma-Aldrich) in the dark at 37°C/3 h. Next, 100 µl of the supernatant was transferred to a new well and the colorimetric change was measured at 492 nm using a microtiter plate reader (Thermo Plate, TP Reader NM).

### Scanning electron microscopy (SEM)

The biofilms were grown on gamma radiation-sterilized and 24 h fetal bovine serum (FBS) (Sigma-Aldrich) pre-treated polystyrene discs measuring 8 mm in diameter and 2 mm in thickness in 24-well plates (TPP) for 60 h at 37°C with agitation. The biofilm method was performed as described above with media and PBS adjusted to 2 mL. First, the biofilms were photographed for visual appearance analysis and, then, fixed in 2.5% glutaraldehyde for 1 h and dehydrated by alcohol solutions with increasing concentrations of ethanol (10%, 25%, 50%, 75% and 90% for 20 min, and 100% for 1 h). The discs were dried at 37°C/24 h (Costa *et al*. 2011). The discs were then transferred to aluminum stubs and covered with gold for 120 s at 40 milliamps (BAL- TEC 50D 050 Sputter Coater) and imaged with Jeol JSM5600.

### Stress susceptibility

The spotting assay was carried out according to the method of Wilson *et al*. (2014) on SD or YPD agar containing 1.5, 2 and 2.5 M NaCl (Roth), 1.5, 2, 2.5 M Sorbitol (Roth), 2 mM H_2_O_2_, 450 µg ml^−1^ Congo red (Sigma-Aldrich), 800 µg ml^−1^ calcofluor white (CFW) (Sigma-Aldrich), 3% ethanol, 0.1%–0.6% SDS (Sigma-Aldrich) and incubated at 37°C for up to 7 days. For UV stress, plates were exposed to 5 mJ of UV-C light using a UV- crosslinker at 254 nm (Bio-Link, Vilber-Lourmat) and incubated at 37°C for up to 7 days. Plates were incubated at 42°C for 4–6 days to assess growth under thermal stress. Heat shock was performed by incubating serial 10-fold dilutions (range 10^6^–10^1^) at 55°C for 10, 15, 20 and 30 min, followed by spotting onto YPD agar plates and incubation at 37°C for up to 7 days. Each experiment was performed at least twice.

### Growth assay

For growth assays under nutrient limitation, agar containing 0.67% YNB plus ammonium sulfate without amino acids (Difco) was supplemented with 2% glucose, 2% potassium acetate, 2% glycerol, 2% galactose, 2% maltose or 2% olive oil as carbon source. For alternative nitrogen sources, agar containing yeast carbon base medium (Difco) was supplemented with 0.5% BSA (Sigma-Aldrich), lysine (Sigma-Aldrich), histidine (Sigma-Aldrich), glycine (Sigma-Aldrich), cysteine (Sigma-Aldrich) or methionine (Sigma-Aldrich) at a concentration of 1 nM. Spot dilution assays (range 10^6^–10^1^) were prepared and plates were incubated at 37°C/3–7 days depending on the carbon and nitrogen source. All experiments were repeated at least twice.

### Filamentation assays

For the filamentation assays, the cells grown overnight in YPD were washed twice in PBS and the cell density was determined using a hemocytometer.

Filamentation on solid media was performed in the following media and incubation times: Spider agar at 30 or 37 °C/7 days; agar supplemented with 10% FBS at 30 or 37°C/7 days; YPS agar [1% yeast extract, 2% bacto-peptone, 2% D(+)-saccharose, 2% agar] seeded by embedding at 25°C/5 days (Chaput *et al*. 1983). The colonies were analyzed visually and the experiments were performed on two different occasions.

For filamentation analysis in liquid media, 5 × 10^4^ fungal cells were added per well in a 12- well cell culture plate containing 10% FBS, YPD, YPD plus 10% FBS, Spider medium, Lee's medium at pH 6.5, RPMI-1640 medium (Invitrogen) or Dulbecco Modified Eagle Medium (DMEM) and the plates incubated at 37°C/3 h, with 5% CO_2_ for RPMI and DMEM. The percentage of filament forms was determined by microscopy observation and the length of the filaments was directly measured using LAS software (Leica Application Suite). The experiments were performed in duplicate and repeated twice.

Induction of filamentation was carried out on oral epithelial cell monolayers (TR146) (Nicholls *et al*. 2009). These cells were infected with 10^5^*C. albicans* cells and incubated at 37°C/3- 6 h with 5% CO_2_ atmosphere. The hyphal cells were then differentially stained according to the invasion assay protocol described below and the length of the hyphae measured using the LAS software (Leica Application Suite). The experiment was performed in triplicate on two different occasions.

For induction of hyphal micro-colonies, approximately 50 fungal cells were added to oral epithelial cell monolayers (TR146) grown according to the invasion assay protocol described below. The plates were incubated at 37°C/24 h in the presence of 5% CO_2_. Next, the cells were fixed with 4% paraformaldehyde and stained with calcofluor white solution. Control micro-colonies were grown on plastic and fixed with 4% paraformaldehyde prior to observation. The dimensions of at least 50 micro-colonies per strain were determined using an inverse microscope (Leica DMIL) and the LAS software (Leica Application Suite). The tests were performed on three different occasions.

Around 100 *Candida* cells were plated on a layer of Spider agar or 10% FBS containing 1.5 or 4% agar and subsequently overlaid with a second layer of medium for invasive growth analysis. Plates were incubated at 30 and 37°C/7 days. The colonies were examined and photographed. The experiment was performed in duplicate on two different occasions.

### Antifungal tests on biofilm

Susceptibility of biofilms to antifungal drugs and growth in the presence of different substances were verified using the method described by Pierce *et al*. (2008). Briefly, overnight YPD cultures were washed and resuspended in pre-warmed RPMI-1640 buffered with MOPS (165 mM, pH 7.0) and adjusted to a concentration of 1 × 10^6^ cells ml^−1^. One hundred microliters of the inocula were added to the well of a 96-well plate and incubated at 37°C/24 h statically. Afterwards, 200 µl of the antifungal solutions in RPMI was added to the washed biofilms and the plates were incubated for additional 24 h at 37°C. The biofilms were exposed to the chemicals over the developing time, 48 h. After the treatments, the biofilm metabolic activity was determined by XTT assay as described above. To determine the quantity of dispersed cells, the plate was shaken in an orbital shaker for 5 min (Solab). Aliquots of serial dilutions from the supernatant were plated onto Sabouraud dextrose agar plates and incubated at 37°C/48 h to determine colony forming units.

The antifungal drugs fluconazole, amphotericin B and caspofungin were used ranging from 2- 1024 mg l^−1^, 0.03125- 16 mg l^−1^, and 0.0625- 32 mg l^−1^, respectively. The biofilms were treated with the chemicals 75 µg ml^−1^ FK506, 20 µM nikkomycin Z, 0.025 M CaCl_2_, 12.5 µg ml^−1^ CFW and 75 µg ml^−1^ Cyclosporin A, all of which were purchased from Sigma-Aldrich and the stock solution prepared in distilled water, except for Cyclosporin A and amphotericin B that were diluted in DMSO (Sigma-Aldrich).

### Oral epithelial cells: adherence, invasion and damage

The human buccal carcinoma epithelial cell lineage TR-146 (Cancer Research Technology, London) (Rupniak *et al*. 1985) was used in the following methods and cultivated as described by Wilson *et al*. (2014).

Invasion rates of the strains were determined and analyzed as previously described by Park *et al*. (2005) and Mayer *et al*. (2012).

For adherence assay, the counting of adhered cells was performed using the slides prepared for the invasion assay at the time point of 3 h. The number of adhered cells was determined by counting 50 high power fields of 150 x 150 µm size on three different occasions.

The method to examine epithelial cell damage infected with *Candida* cells for 24 h has previously been described in detail (Mayer *et al*. 2012).

### Statistical analysis

Differences for the *in vitro* tests and host–pathogen interaction experiments among the strains were analyzed by analysis of variance (ANOVA) and the Tukey test. *P* values < 0.05 were considered to be statistically significant. GraphPad Prism version 6.00 program was used for the statistical analysis.

## RESULTS

### Mrv8 is required for *C. albicans* mycelial micro-colony development and epithelial damage

As part of a larger attempt to characterise unknown function genes in *C. albicans*, we had previously generated a *mrv8*Δ/Δ mutant and found it to exhibit defective epithelial damage (Wilson *et al*. 2014).

We therefore generated a *mrv8*Δ/Δ+*MRV8* complemented strain and analysed the impact of *MRV8* deletion on *Candida*-epithelial interactions in greater detail.

**Figure 1.**
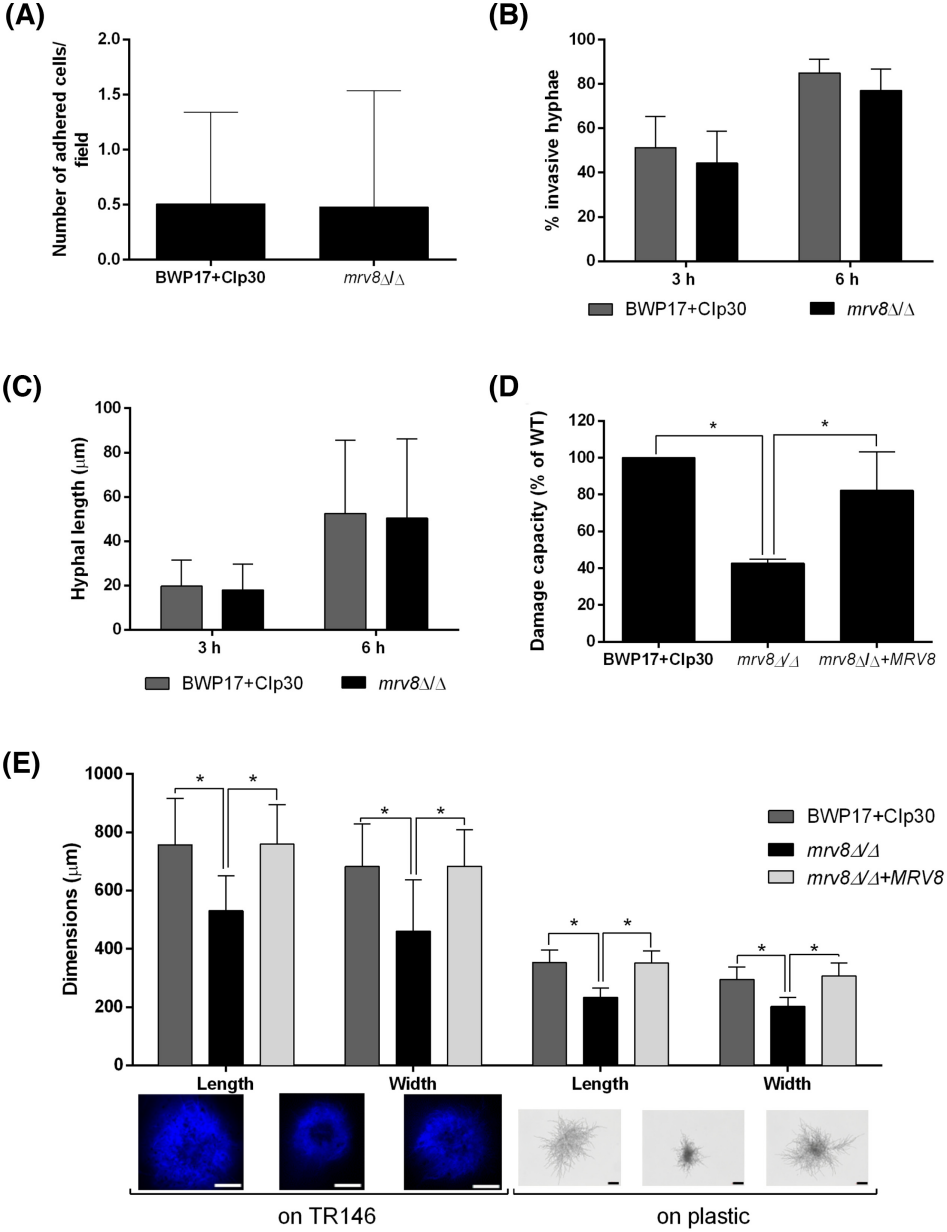
Mrv8 is required for mycelial maturation and damage of oral epithelial cells. Host-pathogen interaction tests were performed on TR146 oral epithelial cell monolayers. **(A)**, *Candida albicans* cells adhered to host cells after 3 h of interaction were counted from 50 fields in three independent experiments. **(B)**, The percentage of invasive hyphae was determined by dividing the number of invaded cells by the total number of cells on epithelia. The results represent the mean ± SD of three independent experiments performed in duplicate. **(C)**, The length of hyphae grown on epithelia for 3 and 6 h was measured using LAS software on two separate occasions carried out in triplicate. **(D)**, Damage capacity was determined for TR146 monolayers infected with *C. albicans* cells for 24 h by measuring lactate dehydrogenase (LDH) levels in the supernatant. Results are the mean ± SD of at least three independent experiments performed in triplicate. Difference statistically significant for multiple comparisons, Tukey's test *P < 0.001. **(E)**, At least 50 microcolonies grown in DMEM medium for 24 h on epithelial cells or on plastic were measured and the results expressed as the mean ± SD of at least three independent experiments. On epithelia, fungi were stained with calcofluor white. Representative pictures of micro-colonies formed by the wild type, mutant and revertant strains, respectively, scale bars: 250 µm on TR146 and 50 µm on plastic. Difference statistically significant for multiple comparisons on TR146 or on plastic, Tukey's test *P < 0.0001.

During the early stages of infection (3 h), the *mrv8*Δ/Δ mutant adhered to and invaded epithelia and formed hyphae at rates almost identical to that of the wild type (Fig. 1A–C). However, at later phases *mrv8*Δ/Δ caused significantly less epithelial damage than the wild type, and complementation with a single copy of *MRV8* restored damage to near wild type levels (Fig. 1D). As this damage defect was unlikely the result of impaired hypha formation, adhesion or invasion during the early stages of the interaction (Fig. 1A–C), we performed morphological analysis of late phase *C. albicans*-epithelial interactions. We infected epithelia with very low numbers of *C. albicans* yeast cells (around 50 per well), and assessed the morphology and dimensions of resultant micro-colonies formed by the different strains. Figure 1E shows that, by 24 h post-infection, deletion of *MRV8* resulted in significantly smaller *C. albicans* micro-colonies on oral epithelia. To test whether this defect was specific for growth on epithelium, we repeated the experiment in cell culture medium on plastic surfaces in the absence of host cells. Again, the *mrv8*Δ/Δ formed smaller micro-colonies than the wild type and complemented strains (Fig. 1E). These data indicate that Mrv8 is dispensable for the initial filamentation, adhesion and invasion events associated with *C. albicans*-epithelial infection, but that the mutant's attenuated damage capacity is likely due to defective mycelial-related growth.

### *Candida albicans* MARVEL family member, Mrv8, is involved in biofilm growth despite showing no other *in vitro* defects

Having confirmed its role in epithelial damage, we next sought to functionally characterise Mrv8. The *mrv8*Δ/Δ mutant was subject to exhaustive functional characterisation tests, revealing no altered phenotype compared to the wild type. *mrv8*Δ/Δ was able to grow at wild type rates on yeast growth media (YNB agar) in the presence of selected sole carbon sources, such as glucose, potassium acetate, glycerol, galactose, maltose or olive oil and on YCB agar in the presence of BSA and the amino acids lysine, histidine, glycine, cysteine, or methionine as sole nitrogen sources. This indicates that Mrv8 is dispensable for yeast phase growth. The *mrv8*Δ/Δ mutant formed filamentous colonies comparable to the wild type on Spider medium and serum-containing agar plates, as well as when embedded in YPS-, Spider- or serum-agar. At the single cell level, the mutant also formed hyphae of similar lengths in liquid hypha-inducing media on a plastic surface, including 10% FBS, YPD plus 10% FBS, Spider medium, Lee's medium at pH 6.5, RPMI-1640 and DMEM with CO_2_. Deletion of *MRV8* also had no obvious impact on the ability of *C. albicans* to grow in the presence of thermal stress, the osmotic stressors NaCl and sorbitol, oxidative stress (H_2_O_2_), the cell wall stressors calcofluor white and Congo Red, and the cell membrane stressors ethanol and SDS or following exposure to UV radiation (data not shown).

**Figure 2.**
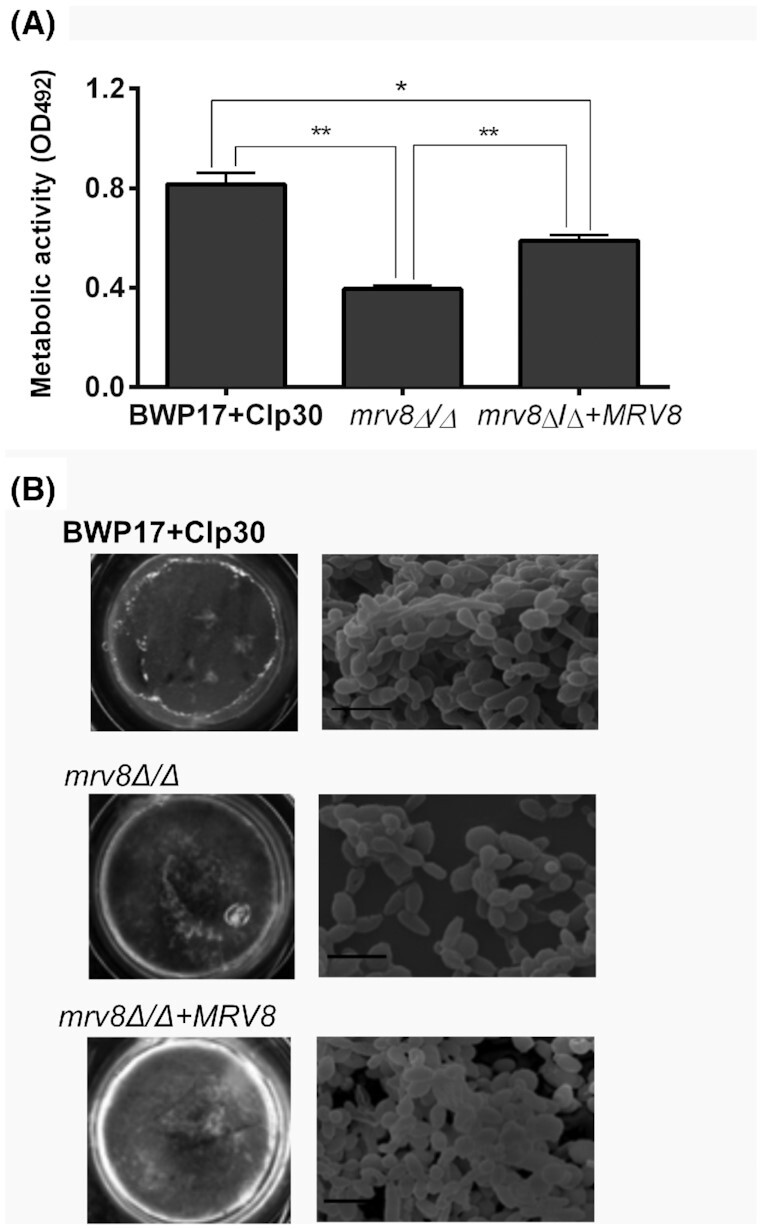
Effect of *MRV8* deletion on *C. albicans* biofilm formation. **(A)**, Metabolic activity of Spider medium biofilms (48 h) as assessed by XTT assay expressed as the mean ± SD of three independent experiments each performed in triplicate. *P < 0.01 and **P < 0.001, Tukey's test. **(B)**, Representative pictures of biofilms grown on polystyrene discs in Spider medium for 60 h and scanning electron microscopy (SEM) of biofilms grown under the same conditions showing a sparse biofilm formed by the *mrv8*Δ/Δ mutant composed of yeasts and pseudohyphae in contrast to the mature biofilm formed by the wild type constituted by multilayers of yeasts, pseudohyphae and hyphae. Scale bars: 7 µm, 5000 x magnification.

When tested for biofilm formation, *mrv8*Δ/Δ exhibited lower metabolic activity in Spider medium (Fig. 2A), but not in RPMI (below). Upon visual inspection, *mrv8*Δ/Δ biofilms appeared aberrant and patchy biofilm compared to the wild type and revertant strains (Fig. 2B). Scanning electron microscopy images revealed a mature wild type biofilm coating the substrate surface and consisting of yeast, pseudohyphae and hyphal cells with evidence of extracellular matrix. In contrast, the *mrv8*Δ/Δ biofilm was sparse and consisted mainly of yeast cells, with little evidence of filamentation. Complementation with a single copy of *MRV8* resulted in an intermediate phenotype: the biofilm covered most of the underlying substrate and all three morphotypes were present (Fig. 2B). Therefore, Mrv8 plays a role in biofilm development in Spider medium.

### *mrv8*Δ/Δ biofilms are less susceptible to caspofungin at higher concentrations

**Figure 3.**
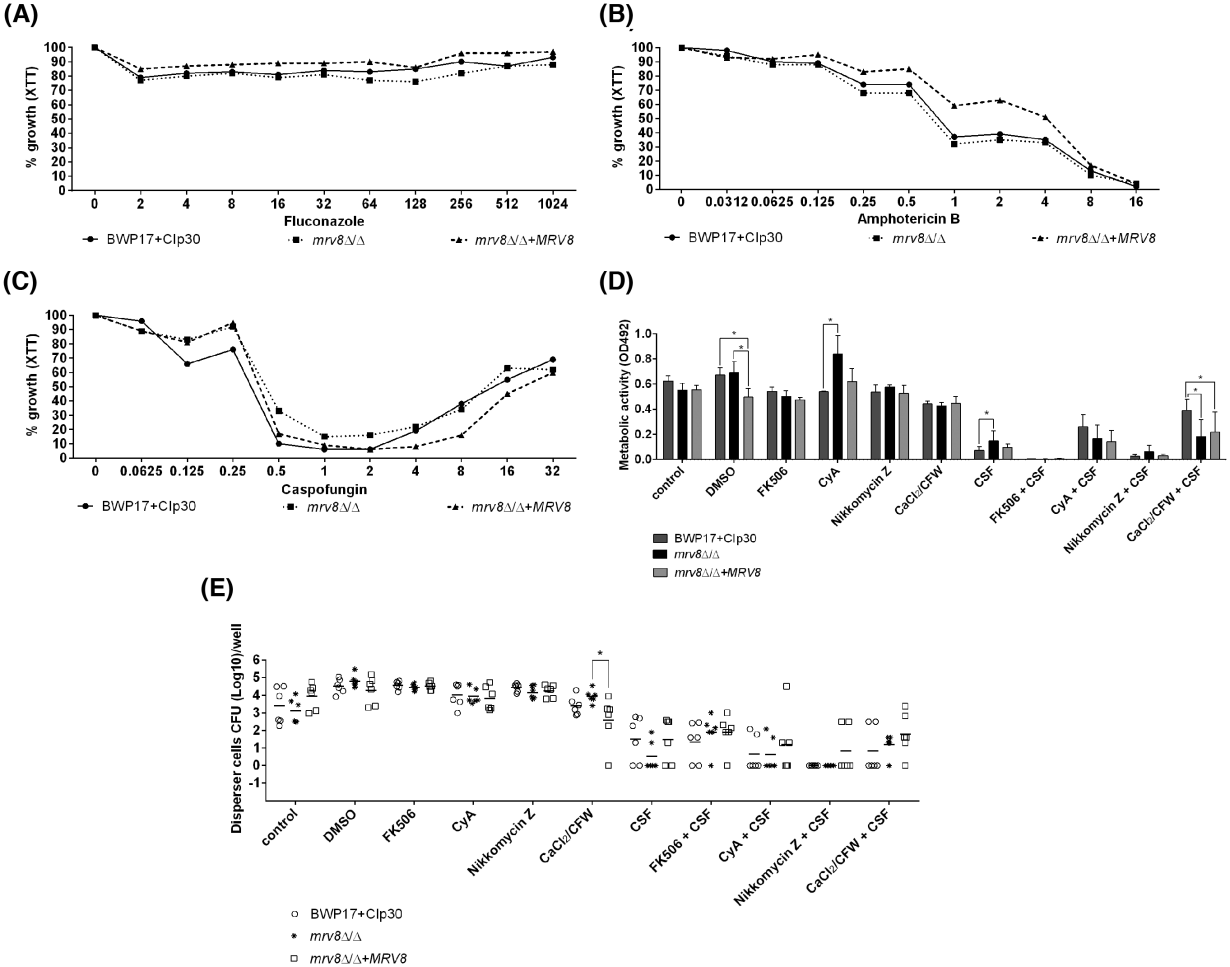
The effect of calcineurin-inhibition and chitin-inhibition and stimulation on Mrv8-dependent biofilm caspofungin tolerance. Biofilms were formed for 24 h and then treated with the antifungal drugs fluconazole (mg l^−1^) **(A)**, amphotericin B (mg l^−1^) **(B)**, or caspofungin (mg l^−1^) (CSF) **(C)** for an additional 24 h. The susceptibility of the biofilms to the drugs was then evaluated by measuring the metabolic activity with XTT-assay. The results are expressed as the percentage of growth in relation to the control (100%). The experiments were performed in triplicate on three different occasions. Alternatively, biofilms were allowed to form for 24 h in the presence of 0.75% DMSO, 75 µg ml^−1^ FK506, 75 µg ml^−1^ cyclosporin A, 20 µM Nikkomycin Z, or 0.025 M CaCl_2_/12.5 µg ml^−1^ CFW before treatment with 0.5 mg l^−1^ caspofungin and the chemicals for a further 24 h. Biofilm metabolic activity (XTT assay **[D]**) or dispersal (CFU (Log_10_)/well **[E]**) was then determined. Results are the mean ± SD of three independent experiments performed in triplicate. Difference statistically significant for multiple comparisons, Tukey's test *P < 0.05.

In order to test the sensitivity of *mrv8*Δ/Δ biofilms to antifungal and chemical treatment, we formed biofilms in RPMI media where, under control conditions, the *mrv8*Δ/Δ mutant behaves similarly to the wild type (Fig. 3).

One of the hallmarks of biofilm physiology is a high tolerance to antifungal drugs. We therefore next questioned whether Mrv8 plays a role in *C. albicans* biofilm antifungal drug resistance. To mimic a therapeutic, rather than prophylactic regime, biofilms were first allowed to develop for 24 h and then treated with different concentrations of antifungals for a further 24 h. Next, biofilm integrity was assessed by XTT assay. Figure 3A and B show that biofilms were fully resistant to fluconazole up to the maximum tested concentration (1024 mg l^−1^) and exhibited dose-responsive inhibition by amphotericin B. Biofilms of all strains exhibited the characteristic eagle effect in the presence of caspofungin, with drug tolerance increasing at higher concentrations. Interestingly, at several drug concentrations (Fig. 3C), biofilms of *mrv8*Δ/Δ exhibited increased caspofungin tolerance. We also tested the sensitivity of planktonic cells to these three drugs and observed no differences amongst the strains (data not shown).

Caspofungin tolerance can be induced by a Ca^2+^/calcineurin -mediated cell wall salvage pathway which causes an increase in chitin content (Munro *et al*. 2007; Walker, Gow and Munro 2013; Rueda, Cuenca-Estrella and Zaragoza 2014). As *MRV8* encodes a predicted plasma membrane protein, we reasoned that the observed differences in caspofungin tolerance in the *mrv8*Δ/Δ mutant may be due to altered Ca^2+^/calcineurin signaling and/or chitin synthesis. In order to test this hypothesis, biofilms were grown for 24 h in the presence of sub-inhibitory concentrations of the calcineurin inhibitors, FK506 and cyclosporin A (CsA), the chitin synthesis inhibitor, Nikkomycin Z or CaCl_2_/CFW (a combination treatment which increases chitin synthesis and caspofungin tolerance) (Walker *et al*. 2008), then treated further with 0.5 mg l^−1^ caspofungin and the chemicals for a further 24 h and biofilm integrity assessed by measuring XTT activity.

Cyclosporin A treatment alone enhanced *mrv8*Δ/Δ biofilm formation compared to the wild type, whilst the other treatments had no effect (Fig. 3D). Pre-treatment of biofilms with cyclosporin A had no significant effect on caspofungin sensitivity whilst both FK506 and Nikkomycin Z increased its antifungal effect. Interestingly, treatment of biofilms with the chitin-stimulatory combination of Ca^2+^/CFW increased caspofungin tolerance in wild type, but not in *mrv8*Δ/Δ biofilms. This is unlikely to be the result of defective chitin synthesis because both chitin and calcium levels were high in *mrv8Δ/Δ* yeast cells following the co-stimulation (Fig. S1, Supporting Information). Indeed, Ca^2+^/CFW-treatment significantly (*P* = 0.0285) enhanced wild type biofilm tolerance against caspofungin by 5.3-fold but had virtually no effect on *mrv8*Δ/Δ biofilms (1.2-fold) and the *mrv8*Δ/Δ+*MRV8* complemented strain exhibited an intermediate (2.3-fold) increase in biofilm activity, which was not significant.

We also determined biofilm dispersal under these different treatment conditions (Fig. 3E). Overall, the level of dispersal reflected biofilm activity as determined by XTT assay (Fig. 3D vs. E). However, whilst caspofungin-treated *mrv8*Δ/Δ biofilms exhibited increased metabolic activity (Fig. 3D), they released fewer disperser cells but this was not statistically significant (Fig. 3E). Together, these data suggested that *mrv8*Δ/Δ biofilms may be associated with altered calcium/calcineurin signaling or chitin synthesis in response to stimulation.

## DISCUSSION

*Candida albicans* is a member of the CTG-Ser Clade of fungi—a group which contains pathogens, non-pathogens, and species with lower virulence than *C. albicans*. Because of this, our group has postulated that lineage-unique genes may contribute to the high virulence potential of *C. albicans*. In a previous study, we deleted a series of lineage unique genes and found several of them to be important for host cell damage. There, we observed a reduction in epithelial and endothelial damage by a *mrv8*Δ/Δ mutant (Wilson *et al*. 2014).

To examine the mechanism underlying this defect, the *mrv8*Δ/Δ and *mrv8*Δ/Δ+*MRV8* strains were evaluated for adhesion, invasion and filamentation in association with epithelia. Complementation of *mrv8*Δ/Δ with a single copy of *MRV8* fully restored epithelial damage, demonstrating that this defect was due to *MRV8* deletion. Adhesion, invasion and initial hypha formation on epithelia was virtually identical for all strains, indicating that early morphogenic events are independent of Mrv8 function. However, at later phases of mycelial development on epithelia, the *mrv8*Δ/Δ mutant exhibited significantly smaller micro-colonies. This was not an epithelial-specific phenomenon, as *mrv8*Δ/Δ micro-colonies were also reduced in size in the absence of host cells. We propose that Mrv8 plays a role in later stages of hyphal development, and the resultant defects in hyphal maturation and ramification may account for the epithelial damage defect of the *mrv8*Δ/Δ mutant. These defects in hyphal maturation may also contribute to the altered biofilm phenotypes observed in this study. The precise mechanism by which Mrv8 contributes to hyphal maturation is currently unknown. Because Mrv8 is a predicted transmembrane protein, it is possible that it acts as a sensor, relaying an extracellular cue to the intracellular signal transduction pathways and transcriptional machinery governing morphogenesis.

Deletion of *MRV8* did not affect biofilm sensitivity to fluconazole or amphotericin B. Interestingly, however, *mrv8*Δ/Δ exhibited increased tolerance to inhibitory concentrations of caspofungin (Fig. 3C).

Caspofungin targets β-1,3-glucan via inhibition of the cognate synthase Fks1 and hot-spot mutations in *FKS1* have been associated with caspofungin resistance (Walker *et al*. 2008; Walker, Gow and Munro 2013). Increased caspofungin tolerance can also be induced by elevations in cell wall chitin (Walker *et al*. 2008). This cell wall salvage pathway is induced by increased *CHS1, CHS3, CHS2*, and *CHS8* expression, elevated chitin synthase activity and increased chitin content in the cell wall in response to caspofungin (Walker *et al*. 2008; Rueda, Cuenca-Estrella and Zaragoza 2014). These events can be mediated through the protein kinase C (PKC), high osmolarity glycerol response (HOG), and Ca^2+^-calcineurin signaling pathways (Walker *et al*. 2008; Walker, Gow and Munro 2013). Calcium stimulation activates the calmodulin-calcineurin pathway and results in Crz1-dependent induction of *CHS2* and *CHS8* (Munro *et al*. 2007). We hypothesized that altered calcineurin signaling may be involved in the caspofungin tolerance phenotype of *mrv8*Δ/Δ mutant biofilms.

In line with this, in the absence of caspofungin, cyclosporin A treatment actually enhanced biofilm metabolic activity for the *mrv8*Δ/Δ mutant suggesting that calcineurin signaling may indeed be altered in this strain.

Exposure to calcium and CFW is known to protect *C. albicans* cells against caspofungin by increasing chitin synthesis (Munro *et al*. 2007; Walker *et al*. 2008; Walker, Gow and Munro 2013; Rueda, Cuenca-Estrella and Zaragoza 2014). We also observed this protective effect with wild type, but not *mrv8*Δ/Δ biofilms. Therefore, in the absence of *MRV8*, CaCl_2_/CFW does not protect *C. albicans* from caspofungin.

As MARVEL proteins are implicated in plasma membrane organization and Mrv8 itself contains four predicted transmembrane helices (Douglas, Wang and Konopka 2013), it is possible that this protein functions at the cell surface, sensing the calcium/CFW signal and possibly signaling through the calcineurin pathway.

In summary, we determined the association of a specific MARVEL family member with pathogenicity and biofilm drug tolerance. *MRV8* was involved in hyphal maturation on both abiotic and epithelial surfaces, damage of epithelial cells, and biofilm-associated caspofungin tolerance, possibly via interactions with calcium- and calcineurin signaling pathways.

## Supplementary Material

foaa033_Supplemental_Files

## References

[bib1] Chandra J , Kuhn DM, Mukherjee PK et al. Biofilm formation by the fungal pathogen *Candida albicans*: development, architecture, and drug resistance. J Bacteriol. 2001;183:5385–94. 11514524 10.1128/JB.183.18.5385-5394.2001 PMC95423

[bib2] Chaput M , Brygier J, Lion Y et al. Potentiation of oxygen toxicity by menadione in *Saccharomyces cerevisiae*. Biochimie. 1983;65:501–12. 6315081 10.1016/s0300-9084(83)80132-1

[bib3] Costa AC , de Campos Rasteiro VM, Pereira CA et al. Susceptibility of *Candida albicans* and *Candida dubliniensis* to erythrosine- and LED-mediated photodynamic therapy. Arch Oral Biol. 2011;56:1299–305. 21704304 10.1016/j.archoralbio.2011.05.013

[bib4] Desai JV , Mitchell AP, Andes DR. Fungal biofilms, drug resistance, and recurrent infection. Cold Spring Harb Perspect Med. 2014;4:501–12. 10.1101/cshperspect.a019729 PMC4200207 25274758

[bib5] Douglas LM , Wang HX, Konopka JB. The MARVEL domain protein Nce102 regulates actin organization and invasive growth of *Candida albicans*. MBio. 2013;4:e00723–13. 24281718 10.1128/mBio.00723-13 PMC3870249

[bib6] Gola S , Martin R, Walther A et al. New modules for PCR-based gene targeting in *Candida albicans*: rapid and efficient gene targeting using 100 bp of flanking homology region. Yeast. 2003;20:1339–47. 14663826 10.1002/yea.1044

[bib7] Gow NA , Hube B. Importance of the *Candida albicans* cell wall during commensalism and infection. Curr Opin Microbiol. 2012;15:406–12. 22609181 10.1016/j.mib.2012.04.005

[bib8] Gulati M , Nobile CJ. *Candida albicans* biofilms: development, regulation, and molecular mechanisms. Microbes Infect. 2016;18:310–21. 26806384 10.1016/j.micinf.2016.01.002 PMC4860025

[bib9] Hawser SP , Douglas LJ. Resistance of *Candida albicans* biofilms to antifungal agents *in vitro*. Antimicrob Agents Chemother. 1995;39:2128–31. 8540729 10.1128/aac.39.9.2128 PMC162894

[bib10] Jin Y , Yip HK, Samaranayake YH et al. Biofilm-forming ability of *Candida albicans* is unlikely to contribute to high levels of oral yeast carriage in cases of human immunodeficiency virus infection. J Clin Microbiol. 2003;41:2961–7. 12843027 10.1128/JCM.41.7.2961-2967.2003 PMC165379

[bib11] Junqueira JC , Fuchs BB, Muhammed M et al. Oral *Candida albicans* isolates from HIV-positive individuals have similar *in vitro* biofilm-forming ability and pathogenicity as invasive *Candida* isolates. BMC Microbiol. 2011;11:247,2180–11-247. 22053894 10.1186/1471-2180-11-247 PMC3217868

[bib12] Kuhn DM , George T, Chandra J et al. Antifungal susceptibility of *Candida biofilms*: unique efficacy of amphotericin B lipid formulations and echinocandins. Antimicrob Agents Chemother. 2002;46:1773–80. 12019089 10.1128/AAC.46.6.1773-1780.2002 PMC127206

[bib13] Lazzell AL , Chaturvedi AK, Pierce CG et al. Treatment and prevention of *Candida albicans* biofilms with caspofungin in a novel central venous catheter murine model of candidiasis. J Antimicrob Chemother. 2009;64:567–70. 19584104 10.1093/jac/dkp242

[bib14] Martin R , Moran GP, Jacobsen ID et al. The *Candida albicans*-specific gene EED1 encodes a key regulator of hyphal extension. PLoS One. 2011;6:e18394. 21512583 10.1371/journal.pone.0018394 PMC3075580

[bib15] Mathe L , Van Dijck P. Recent insights into *Candida albicans* biofilm resistance mechanisms. Curr Genet. 2013;59:251–64. 23974350 10.1007/s00294-013-0400-3 PMC3824241

[bib16] Mayer FL , Wilson D, Jacobsen ID et al. The novel *Candida albicans* transporter Dur31 Is a multi-stage pathogenicity factor. PLoS Pathog. 2012;8:e1002592. 22438810 10.1371/journal.ppat.1002592 PMC3305457

[bib17] Moyes DL , Wilson D, Richardson JP et al. Candidalysin is a fungal peptide toxin critical for mucosal infection. Nature. 2016;532:64–8. 27027296 10.1038/nature17625 PMC4851236

[bib18] Munro CA , Selvaggini S, de Bruijn I et al. The PKC, HOG and Ca2+ signalling pathways co-ordinately regulate chitin synthesis in *Candida albicans*. Mol Microbiol. 2007;63:1399–413. 17302816 10.1111/j.1365-2958.2007.05588.x PMC2649417

[bib19] Nett J , Lincoln L, Marchillo K et al. Putative role of beta-1,3 glucans in *Candida albicans* biofilm resistance. Antimicrob Agents Chemother. 2007;51:510–20. 17130296 10.1128/AAC.01056-06 PMC1797745

[bib20] Nett JE , Sanchez H, Cain MT et al. Interface of *Candida albicans* biofilm matrix-associated drug resistance and cell wall integrity regulation. Eukaryot Cell. 2011;10:1660–9. 21666076 10.1128/EC.05126-11 PMC3232725

[bib21] Nicholls S , Leach MD, Priest CL et al. Role of the heat shock transcription factor, Hsf1, in a major fungal pathogen that is obligately associated with warm-blooded animals. Mol Microbiol. 2009;74:844–61. 19818013 10.1111/j.1365-2958.2009.06883.x PMC3675641

[bib22] Nobile CJ , Johnson AD. *Candida albicans* Biofilms and Human Disease. Annu Rev Microbiol. 2015;69:71–92. 26488273 10.1146/annurev-micro-091014-104330 PMC4930275

[bib23] Nobile CJ , Mitchell AP. Regulation of cell-surface genes and biofilm formation by the *C. albicans* transcription factor Bcr1p. Curr Biol. 2005;15:1150–5. 15964282 10.1016/j.cub.2005.05.047

[bib24] Park H , Myers CL, Sheppard DC et al. Role of the fungal Ras-protein kinase A pathway in governing epithelial cell interactions during oropharyngeal candidiasis. Cell Microbiol. 2005;7:499–510. 15760450 10.1111/j.1462-5822.2004.00476.x

[bib25] Pierce CG , Uppuluri P, Tristan AR et al. A simple and reproducible 96-well plate-based method for the formation of fungal biofilms and its application to antifungal susceptibility testing. Nat Protoc. 2008;3:1494–500. 18772877 10.1038/nport.2008.141 PMC2741160

[bib27] Ramage G , Rajendran R, Sherry L et al. Fungal biofilm resistance. Int J Microbiol. 2012;2012:528521. 22518145 10.1155/2012/528521 PMC3299327

[bib26] Ramage G , Robertson SN, Williams C. Strength in numbers: antifungal strategies against fungal biofilms. Int J Antimicrob Agents. 2014;43:114–20. 24359842 10.1016/j.ijantimicag.2013.10.023

[bib28] Robbins N , Uppuluri P, Nett J et al. Hsp90 governs dispersion and drug resistance of fungal biofilms. PLoS Pathog. 2011;7:e1002257. 21931556 10.1371/journal.ppat.1002257 PMC3169563

[bib29] Rueda C , Cuenca-Estrella M, Zaragoza O. Paradoxical growth of *Candida albicans* in the presence of caspofungin is associated with multiple cell wall rearrangements and decreased virulence. Antimicrob Agents Chemother. 2014;58:1071–83. 24295973 10.1128/AAC.00946-13 PMC3910852

[bib30] Rupniak HT , Rowlatt C, Lane EB et al. Characteristics of four new human cell lines derived from squamous cell carcinomas of the head and neck. J Natl Cancer Inst. 1985;75:621–35. 2413234

[bib31] Singh SD , Robbins N, Zaas AK et al. Hsp90 governs echinocandin resistance in the pathogenic yeast *Candida albicans* via calcineurin. PLoS Pathog. 2009;5:e1000532. 19649312 10.1371/journal.ppat.1000532 PMC2712069

[bib32] Soll DR , Daniels KJ. Plasticity of *Candida albicans* Biofilms. Microbiol Mol Biol Rev. 2016;80:565–95. 27250770 10.1128/MMBR.00068-15 PMC4981664

[bib33] Taff HT , Nett JE, Zarnowski R et al. A *Candida* biofilm-induced pathway for matrix glucan delivery: implications for drug resistance. PLoS Pathog. 2012;8:e1002848. 22876186 10.1371/journal.ppat.1002848 PMC3410897

[bib34] Tobudic S , Kratzer C, Lassnigg A et al. Antifungal susceptibility of *Candida albicans* in biofilms. Mycoses. 2012;55:199–204. 21793943 10.1111/j.1439-0507.2011.02076.x

[bib35] Uppuluri P , Srinivasan A, Ramasubramanian A et al. Effects of fluconazole, amphotericin B, and caspofungin on *Candida albicans* biofilms under conditions of flow and on biofilm dispersion. Antimicrob Agents Chemother. 2011;55:3591–3. 21518839 10.1128/AAC.01701-10 PMC3122381

[bib36] Walker LA , Gow NA, Munro CA. Elevated chitin content reduces the susceptibility of *Candida* species to caspofungin. Antimicrob Agents Chemother. 2013;57:146–54. 23089748 10.1128/AAC.01486-12 PMC3535899

[bib37] Walker LA , Munro CA, de Bruijn I et al. Stimulation of chitin synthesis rescues *Candida albicans* from echinocandins. PLoS Pathog. 2008;4:e1000040. 18389063 10.1371/journal.ppat.1000040 PMC2271054

[bib38] Wilson D , Mayer FL, Miramon P et al. Distinct roles of *Candida albicans*-specific genes in host-pathogen interactions. Eukaryot Cell. 2014;13:977–89. 24610660 10.1128/EC.00051-14 PMC4135803

