# Supplementary figures and images for: Candida albicans Mrv8, is involved in epithelial damage and biofilm formation
Source: FEMS Yeast Res. 2020 Jun 25;20(5):foaa033. doi: 10.1093/femsyr/foaa033 (PMC7343537; doi:10.1093/femsyr/foaa033)

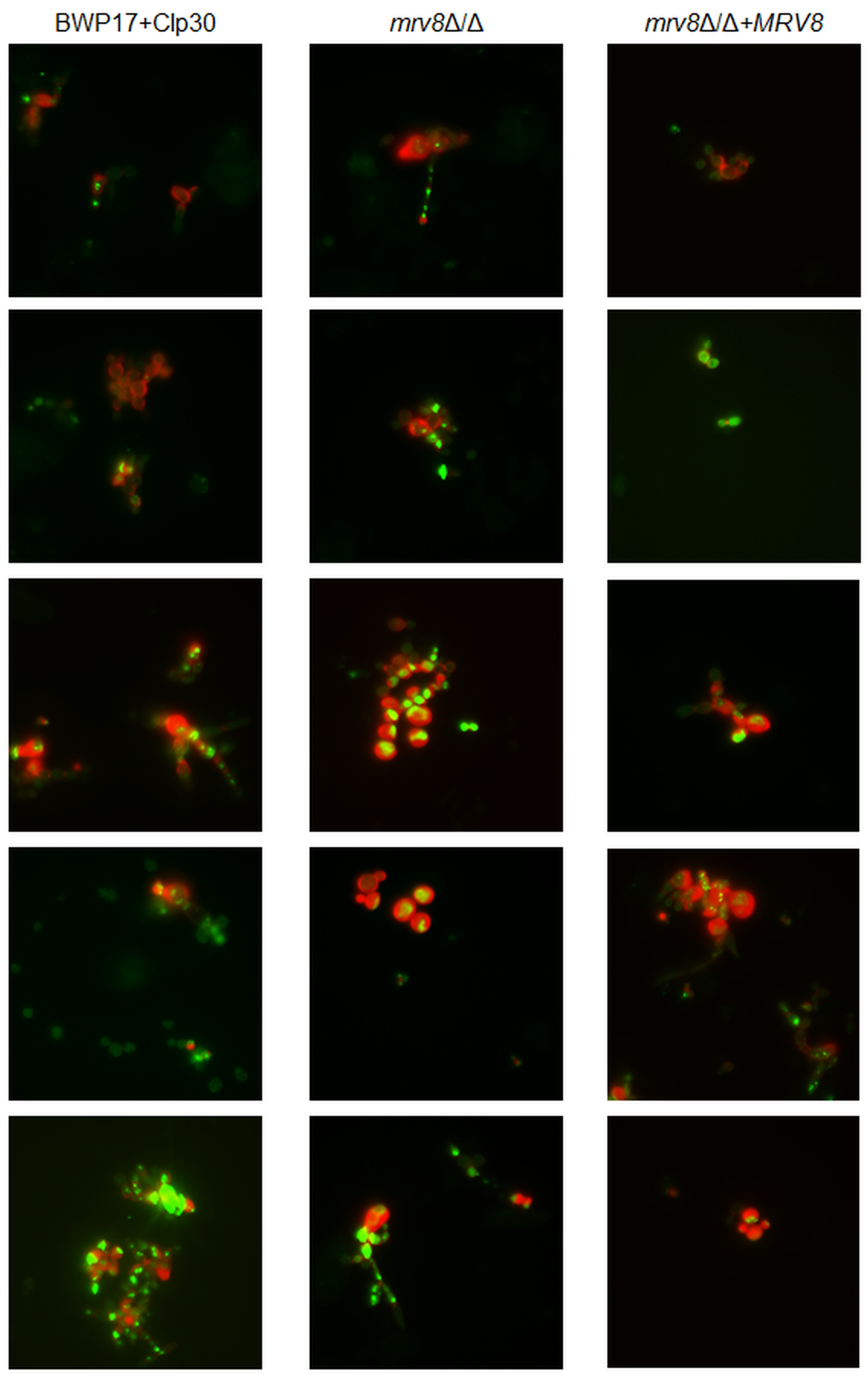

Supplement: foaa033_Supplemental_Files [file foaa033_supplemental_files.zip › Supplementary_Figure_1-revised_manuscript.tif]
