# Supplementary material for: Candida albicans Mrv8, is involved in epithelial damage and biofilm formation
Source: FEMS Yeast Res. 2020 Jun 25;20(5):foaa033. doi: 10.1093/femsyr/foaa033 (PMC7343537; doi:10.1093/femsyr/foaa033)
Supplement: foaa033_Supplemental_Files [file foaa033_supplemental_files.zip › Supplementary_Figure_1-revised_manuscript.doc]

**Supplementary data**

**Supplementary Figure 1.** Representative pictures of overlaid images (red= CFW fluorescence, green= calcium intracellular fluorescence) of the wild type, *mrv8*Δ/Δ, and *mrv8*Δ/Δ::*MRV8* grown in YPD + 0.2 M CaCl_2_/ 100 µM CFW stained with Oregon Green 488 BAPTA-1.
